# LIFE AT AGE 100 IN SWITZERLAND: FINDINGS FROM THE SWISS100 CENTENARIAN STUDY

**DOI:** 10.1093/geroni/igae098.0215

**Published:** 2024-12-31

**Authors:** Daniela Jopp

**Affiliations:** Chair

## Abstract

Switzerland is among the countries with the highest life expectancy and has seen a strong increase of very old individuals over the past century. Yet very little is known about its oldest-old population, including those aged 100 and older. This symposium presents findings from the first national centenarian study conducted in Switzerland, SWISS100. The presentations offer findings from the SWISS100 Main Study, allowing due to its interdisciplinary nature a comprehensive perspective on life at age 100 in Switzerland. Jopp and colleagues will present findings on key characteristics in centenarians (e.g., health, cognition, social resources, psychological strengths) and their associations with quality of life. Stahlmann and colleagues will share findings on the use of ambulatory assessment in centenarians, more specifically sound recordings which allow to investigate everyday life activities, showing that such innovative tools are applicable in this population and that these methods lead to important insights, complementing self-report. Masotti and colleagues will present results on the caregiving setting in centenarians, illustrating that Swiss centenarians show a notable variation in needs and support. Hoffman and colleagues will present results on centenarians’ goals, coded by content and orientation, and associations to well-being outcomes, illustrating that goals remain important adaptation features in very old age. Overall, SWISS100 findings help to better understand the specific life phase centenarians are experiencing, including its challenges, but also show notable psychological resilience and well-being.

